# Magnitude of patient satisfaction and its associated factors at the outpatient psychiatry service of Dilla university referral hospital, Southern Ethiopia, Dilla, 2020

**DOI:** 10.1371/journal.pone.0272485

**Published:** 2022-08-03

**Authors:** Chalachew kassaw, Alem Eskeziya, Tamrat Anbesaw

**Affiliations:** 1 Department of Psychiatry, College of Health Science, Dilla University, Dilla, Ethiopia; 2 Department of Psychiatry, College of Medicine and Health Science, Wollo University, Dessie, Ethiopia; PLOS: Public Library of Science, UNITED KINGDOM

## Abstract

**Background:**

Patient satisfaction is a subjective attitudinal response of a client to a health institution’s services and a pillar of quality assurance. Patients who are happy with their treatment are much more likely to stick with it, improve quickly, and function normally. Satisfied patients are more compliant, improve faster, and are more functional. However, there haven’t been enough studies conducted across the country, and none have been conducted in this study area. As a result, the purpose of the study was to estimate the size of patient satisfaction and associated determinants at Dilla University Referral Hospital’s psychiatry unit in Dilla, 2020.

**Methods:**

This was a hospital-based cross-sectional study design utilized using a simple random sampling technique. To assess patient satisfaction, we used the 24-item Mental Health Service Satisfaction Scale which was a validated tool in Ethiopia. The link between the outcome and the independent variable was determined using linear regression analysis (P< 0.05).

**Result:**

This study enrolled 409 respondents with a response rate of 97%. The overall mean percentage score of patient satisfaction was 55.4% (95% CI (48.4%– 59.2%). Having bipolar disorder diagnosis [β = -2.93, 95% CI (-4.33, -1.96), p = .000], distance from the hospital [β = -2.34), 95% CI (-3.765, -1.735), P = .001], waiting time [β = -2.19, 95% CI (-3.49, -1.10), p = .000], monthly income (2.95, 95% CI (1.65, 5.23) and Urban residence (β = 1.43, 95% CI (1.03–3.43), p = 0.01) were variables significantly associated with perceived patient satisfaction.

**Conclusions and recommendations:**

In this study, more than half of the respondents scored above the mean percentage score of patient satisfaction. The amount of time spent in the waiting area and the distance traveled to the hospital were identified as variables that could be improved by working with different stakeholders.

## Introduction

Satisfaction is the fulfillment of an individual’s expectations, wants, and needs in relation to something. It serves as a foundation for quality control, subjective responses, and emotionally charged situations [1, 2]. Due to the dynamic nature of needs, expectations, and service settings, there is no universally approved measuring tool for patient satisfaction [3]. Previously, mentally ill patient attendants were the primary focal persons in studies of service satisfaction at mental health hospitals, but more recently, psychiatric patients have been included in research to improve the quality of the service [4]. Only 0.00113 percent of Ethiopians visited outpatient psychiatry clinics, although more than half of those who did have at least one psychotropic drug from each medication group [5, 6]. The World Health Organization recommends that health institutions provide client-centered services in order to improve the quality of care [7]. In comparison, to low-income countries, patient satisfaction is relatively better in developed countries [8]. In Ethiopia, patient satisfaction in outpatient psychiatry services ranges from 50.3% (Jimma) [9] to 77% (Gondar) [10]. Service and clinical considerations, such as the affordability of service and the patient’s clinical response, were the most important elements influencing client satisfaction [11–13]. For quality assurance, better therapeutic results, and professional burnout, assessing service satisfaction on a regular basis is critical. Clients who were happy with the service had fewer readmissions, faster follow-up, and fewer suicide attempts [14, 15]. Several researchers and healthcare system organizations have indicated that delivering psychoeducation about the therapy, adding community service, staff training, and adhering to set guidelines are crucial pillars to increase patient satisfaction [16, 17]. Mental mental health services have been provided in most parts of the country over the past 30 years, and patient service satisfaction is a reliable indicator of quality health care. However, taking measurements directly from psychiatric patients is uncommon. Furthermore, no research has been done in this area. As a result, the goal of this study was to determine the magnitude of patient satisfaction and its associated factors at Dilla University Referral Hospital, psychiatry clinic, 2020.

## Methods and materials

### Study area, period, and design

An institutional-based cross-sectional study was conducted at Dilla university referral hospital, psychiatric clinic from April 12 to May 12, 2020. The hospital is located in the southern part of Ethiopia. The psychiatry service has been begun in 1978 G.C, and monthly around 530 patients were attending their mental health service.

### Eligibility criteria

#### Inclusion criteria

- All mentally ill patients age 18 and above years old.

#### Exclusion criteria

- All mentally ill patients with < 6 months duration of the visit.
- Respondents with acute or severe psychiatry conditions.

### Sampling technique and sample size determination

Since the outcome variable of this study was continuous, to calculate the sample size, we used the percentage mean satisfaction scores of a study conducted in Jimma medical center (50.3%) [9].

The formula was

$$\begin{array}{ll} \text{n} & =\frac{{\left(\text{Z}\;\alpha /2\right)}^{2}\;\text{P}\;\left(1‐\text{P}\right)}{{\text{d}}^{2}}\\ & =\left(1.96\right)\;\left(1.96\right)\;\left(0.497\right)\;\left(0.503\right)\;/\;\left(0.05\right)\;\left(0.05\right)\\ & =384 \end{array}$$

Where, n = required sample size

p = 0.503

q = 0.497

z(α/2) = is reliability coefficient at 95% confidence interval (1.96)

d = (margin of error) = 0.05

N = non-response rate = 10%.

The total sample size was, 384+ 38.4 = 422.

#### Sampling technique

This study used a sampling frame of the total number of respondents (530) who came for follow-up in one month, and a computerized produced approach to pick the required number of study samples (422).

### Data collection instrument

#### Socio-demographic related factors

A semi-structured questionnaire was used which has different subunits, questionnaires to assess socio-demographic factors, patient clinical characteristics, and hospital service-related factors.

The instruments used for the data collection were the following validated assessment tools.

**The mental health satisfaction scale (MHSSS)**- It’s a 24-item questionnaire that’s used to gauge patient satisfaction with mental health services. Responses are categorized as (1) for strongly disagree, (2) disagree, (3) agree, and (4) strongly agree for each of the 24 items. The range of scores was 24 (minimum score) to 96 (maximum score), and because there was no cut-point or continuous variable, each respondent’s actual score was reported as the sum total of each questioner’s items. The tool was validated in Ethiopia with Cronbach alpha (α = 0.84) [18].

**Clinical Global Impressions-Severity (CGI-S)**–It’s a seven-item scale based on physicians’ experience analyzing current clinical status and improvement to determine the clinical severity of psychiatric illnesses. Each of the three components is given a score ranging from 0 to 7. The Cronbach alpha of this tool was (0.78) [19].

**The Alcohol, Smoking, and Substance Involvement Screening Test (ASSIST-3.0)**—It was used to determine the subjects’ current and past usage of alcohol, cigarettes, khat, and cannabis. A lifelong user is someone who has used any substance throughout their life, whereas a current substance user is someone who has used any substance during the last three months. The Cronbach alpha of this tool was (0.80) [19].

### Data collection procedures

This study used a face-to-face interview and a document review to collect data from each of the respondents. Face-to-face interviews were utilized to examine socio-demographic and service-related characteristics, whereas document interviews were used to assess clinical diagnosis and illness severity. Data were collected by four data collectors with a bachelor’s degree in nursing and two master’s level mental health specialist supervisors. The data collectors and supervisors were given three days of training about the study’s objective, methods, and ethical concerns prior to the actual data collection period. Each data collector employed 30–45 minutes for both interviews and card reviews to fill and complete a single respondent questionnaire. The principal investigator and supervisor checked the completeness of each collected data on each day of data collection and discarded all of the incomplete questionnaires.

### Study variables

#### Dependent variable

Patient satisfaction.

#### Independent variables

**Socio-demographic related factors**:- Age, Gender, Educational status, Marital status, Place of residency, and Monthly Income.

**Patient clinical characteristics**:- Current substance use, Current clinical diagnosis, Duration of illness, Comorbid medical illness, and Clinical severity scale.

**Hospital service-related factors**:- Waiting time, Consultation time, Accessibility of service, and Distance from the hospital.

### Operational definitions

**Percentage mean score patient satisfaction**: (actual score—potential minimum score)/ (potential maximum—potential minimum) ×100% = (P1%+P2%+....+P423%). Where, P-represents participants [20].

**Waiting time**: The total time from registration until consultation with a doctor which was measured by recording the time of arrival to consultation by the data collector [21].

**Consultation time**: it is the time which a patient talks with a clinician about his treatment and is recorded by the data collector from entry to exit of the consultation office.

**Co-morbid medical illness**: if the patient had proven or diagnosed medical illness which was determined by reviewing the patient chart.

**Prior trial of treatment**: The first trial of treatment when the patient becomes mentally ill either it could be traditional or modern.

**Family involvement in treatment**: Any individuals who are related to the patient through marriage, biology or adoption, friendship and involve in the patients treatment process like encourage engagement with treatment plans, recognize and respond to early warning signs of relapse, assist in accessing services during a period of crisis, giving medication, attending for follow up with a patient, asking clinicians about the possible solutions of disorder and deciding about some issues regarding the treatment which was assessed by yes or no question [22].

**Current substance user**: a respondent who use any of psychoactive substances within recent 3 months [23].

**Courtesy**: It refers to health care providers respect and politeness shown to the patients (e.g. doctors, nurse and pharmacy assistants, and other healthcare personnel).

**Long duration of illness**: patients who had more than 5 years ‘ duration of illness.

**Social support**: OSS -3 score of 3–8 = poor social support, 9–11 = moderate social support and 12–14 strong social support [24].

**The severity of the current psychiatric illness**: It was assessed based of clinical experience and the current condition of the patient score.

0 = **(Not assessed)**

1 = (**Normal, not at all ill)**: Symptoms are rarely present and occur only in contextually appropriate circumstances. The patient reports functioning at or very close to their full capacity

2 = (**Borderline mentally ill)**: Symptoms are few in number and only intermittently present, and usually no more than mild severity. There is little or no interference in role functioning.

3 = (**Mildly ill)**: Symptoms are clearly present and cause distress, but there is only minimal or no reduction in functioning

4 = **(Moderately ill)**: Symptoms are present every day or nearly every day but may diminish at times. Substantial distress is present but bearable. Functioning in important roles is somewhat reduced, or maintained only through high levels of perceived effort. Suicidal thoughts may be present, but there is usually a desire to live.

5 = (**Markedly ill)**: Symptoms are highly distressing and the patient struggles greatly to function in important life roles. Active suicidal ideation may be present

6 = (**Severely ill**): Symptoms are nearly constant and highly distressing, and the patient is unable to function in important life roles. Active suicidal ideation may be present

7 = (**Extremely ill)**: Symptoms are continuously present at a very severe level. The person is unable to maintain basic functioning. Active suicidal thoughts are usually present. Hospitalization is usually required [25].

### Data analysis

To minimize the data entry error, the coded data were entered into EPI-DATA version 3.1 and then exported to SPSS version 22. 00 for analysis. Due to the continuous nature of the outcome variable, simple linear regression analysis was used to assess the statistical relationship between dependent and independent variables. A simple linear regression analysis model at P< 0.25 was used to identify variables candidates for multiple linear regressions. Multiple linear regression analysis at a 95% Confidence interval and P< 0.05 was used to adjust the confounders and determine the independent predictors of the outcome variable. The assumption of linear regression, such as normality, linearity, outlier, and, multicollinearity was checked and met before performing linear regression analysis.

### Data quality assurance

The English version questionnaire was translated to the local languages such as Gedeoffa and Amharic using two different translators and back-translated to English for consistency and check-up. Before the actual data collection, The pretest was done on the 5% of the participants at Yirgachife general hospital psychiatric outpatient clinic to check for the reliability, understand-ability, and clarity of the questionnaire The internal consistency of this mental health service satisfaction tool in the pretest study was (Cronbach’s Alpha = 0.85).

### Ethical consideration

Before data collection, and ethical clearance letter (DU-303/20) was obtained from the Institutional Review Board (IRB) of, Dilla University college of medicine and health. Written informed consent was obtained from each of the participants before the data collection. The information was kept confidential. Permission to conduct the research was obtained from the clinical director of the hospital and the head of the Psychiatric Clinic. The participants were assured that they had the right to withdraw from the interview at any time they wish. And they were ascertained that if they wish to refuse to participate, their care or dignity had not been compromised in any way since there is no relationship between participation and the health service they received. Participants were informed that there is no expectation of additional treatment or any associated benefits and risks for them participating in the study. Finally, the questionary was locked after data entry was completed.

## Result

### Socio-demographic characteristics of respondents

This study included 409 respondents with a 97% response rate. Among the total respondents, 266 (65%) were males, and the Mean ± SD age of respondents was 30±7, which ranges from 18 to 75 years old. More than half of the respondents, 233 (57%) were protestant religion followers. The mean ± SD monthly income of the respondents was 368 ± 220 ethiopian birr (ETB). More than half of the respondents, 261(63.9%), came from an urban and median distance of respondents from the hospital was 35.3 (Min = 1, Max = 300) km. Almost all of the respondents, 396 (96.8%) had no health insurance (Table 1).

**Table 1:** Socio-demographic characteristics of respondents at Dilla university referral hospital, southern Ethiopia, Dilla, 2020 (n = 409).

Variable		Frequency (N = 409)	Percent (%)
Sex	Male	266	65%
	Female	143	35%
Religion	Protestant	233	57%
	Orthodox	143	35%
	Muslim	33	8%
Marital status	Married	163	39.8%
	Single	210	51.3%
	Divorced	36	0.88%
Educational status	No education	31	7.6%
	Primary	167	40.8%
	Secondary	95	23.2%
	More than secondary	116	28.4%
Occupation	Student	28	6.8%
	House wife	39	9.5%
	Merchant	56	13.7%
	Government employee	87	21.3%
	Farmer	74	18.1%
	Private work	84	20.5%
	Jobless	41	10.02%
Residency	Urban	261	63.9%
	Rural	148	36.1%
Health insurance	Yes	396	96.8.8%
	No	13	3.2%

### Clinical related factors of respondents

The mean ± SD of total duration and age onset of illness was 6± 5 years, and 27 ± 7 years, respectively. The mean ± SD of waiting and consultation time reported was 56± 25 and 14 ± 5 minutes, respectively. Nearly half of the respondents, 211 (51.6%) had a history of admission. Nearly one -thirds of the respondents, 145 (35.5%) were attending traditional treatment. Out of all respondents, 237 (57.9%) of them had no current history of substance use (Table 2).

**Table 2:** The clinical and service-related factors of respondents at Dilla university referral hospital, southern Ethiopia, Dilla, 2020 (n = 409).

Variable	Category	Frequency	Percent(%)
Having co morbid medical illness	Yes	23	5.62%
	No	386	94.3%
Severity of the illness	Normal, not at all	49	12%
	Borderline mentally ill	311	76.0%
	Mildly ill	49	12%
Social support scale	Poor	235	57.5%
	Moderate	149	36.4%
	Strong	25	6.1%
Current Substance use history	Yes	172	42.1%
	No	237	57.9%
History of admission	Yes	211	51.6%
	No	198	48.4%
First trail treatment	Modern	264	64.5%
	Traditional	145	35.5%

### Clinical diagnosis of the respondents

**Fig 1:**
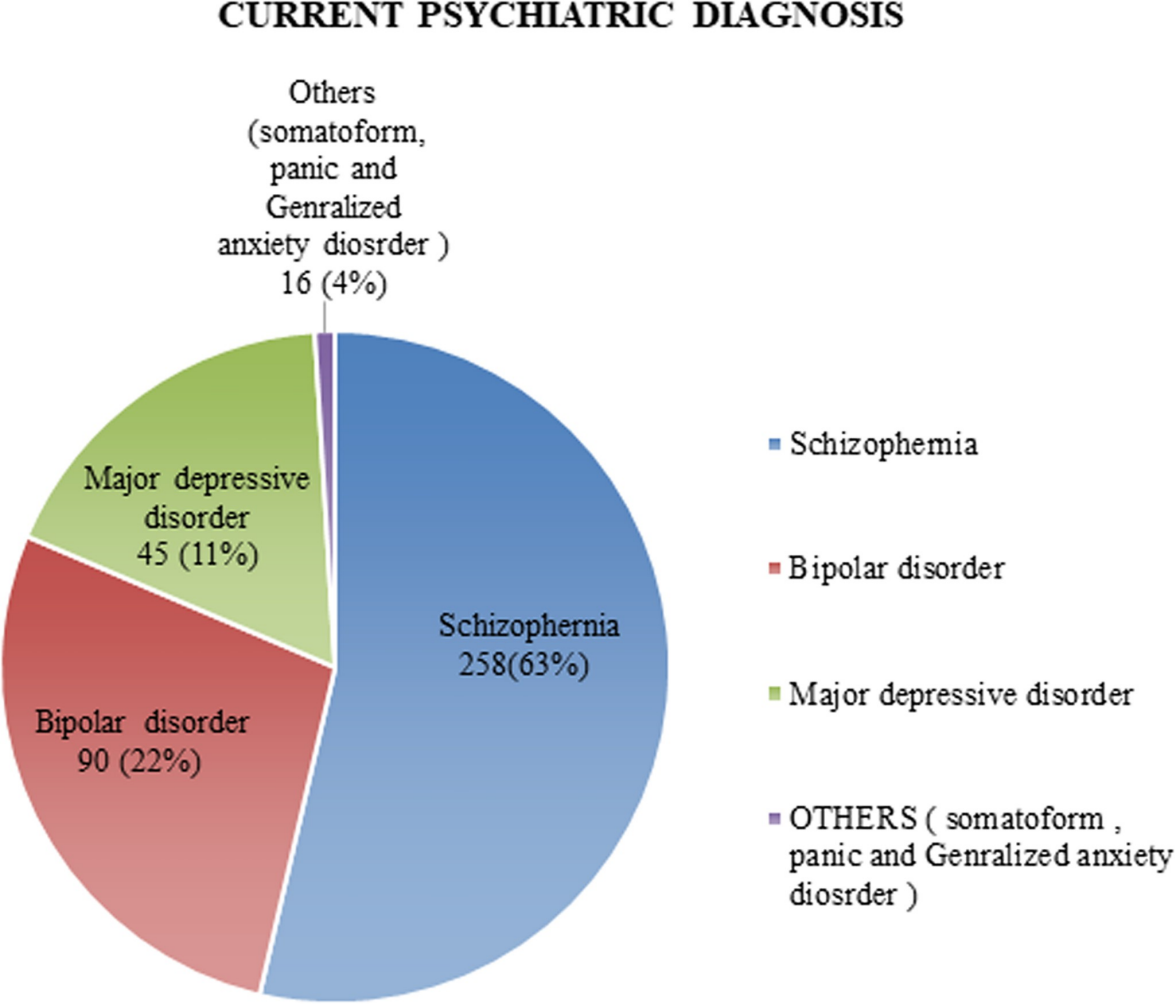
The current psychiatry diagnosis of respondents at Dilla university referral hospital, southern Ethiopia, Dilla, 2020 (n = 409).

From all respondents, nearly two-thirds of the respondents, 258(63%) had been diagnosed with schizophrenia (Fig 1).

### Mental health service satisfaction item response of the respondents

Out of all items used to measure service satisfaction, the majority of the respondents reported strongly disagreeing with the items asking about affordability, chance to be followed by the same professional, is not helping to prevent relapse, no way to be visited by specialists, and not getting enough information about the (Table 3).

**Table 3:** The response of respondents for each mental health service satisfaction assessment item at Dilla university referral hospital, Southern Ethiopia, 2020, (n = 409).

Items	Strongly disagree	Disagree	Agree	Strongly agree
The health worker treated me with courtesy	33(8.1%)	45(11.1%)	22(5.37%0	309(75.5%)
The health worker listened to me carefully	12(2.93%)	19(4.64%)	134(32.7%)	244(59.6%)
The health worker explained things to me in a way I understood	29(7%)	57(13.9%)	125(30.5%)	198(48.4%)
The health facility was clean	76(18.5%)	67(16.3%)	124(30.3%)	142(34.7%)
The waiting room was clean	54(13.2%)	79(19.3%)	143(34.9%)	133(32.5%)
The latrine was clean	162(39.6%)	59(14.4%)	96(23.4%)	92(22.4%)
The waiting time was acceptable	124(30.3%)	94(22.9%)	73(17.8%)	118(28.8%)
I had enough time to discuss with health worker	85(20.7%)	64(15.6%)	179(43.7%)	81(19.8%)
I was given information in a way I understood	189(46.2%)	78(19%)	65(15.8%)	77(18.8%)
I received helpful advice	41(10%)	36(8.8%)	156(38.1%)	176(43%)
The administrative staff treated me with courtesy and respect	67(16.3%)	39(9.5%)	124(30.3%)	179(43.7%)
The health worker involved my family helpfully	125(30.5%)	59(14.4%)	76(18.5%)	149(36.4%)
My privacy was respected	110(26.8%)	78 (19%)	68(16.6%)	153(37.4%)
I have the opportunity for follow up with the same health worker	219(53.5%)	56(13.6%)	69(16.8%)	65(15.8%)
My personal information is kept confidential	35(8.5%)	72(17.6%)	179(43.7%)	123(30%)
Referral to specialist is possible	256(62.5%)	56(13.6%)	53(12.9%)	44(10.7%)
The service is effective at decreasing symptoms	60(14.6%)	57(13.9%)	143(34.9%)	149(36.4%)
The service is effective at decreasing relapses	75(18.3%)	184(44.9%)	78(19%)	72(17.6%)
The service is effective at helping with economic problems	266(65%)	47(11.4%)	53(12.9%)	43(10.5%)
It is possible to see the health worker when needed	23(5.6%)	51(12.4%)	168(41%)	167(40.8%)
It was easy to attend the health facility	60(14.6%)	56(13.6%)	231(56.4%)	62(15.1%)
I had enough time to attend the health facility	56(13.6%)	59(14.4%)	251(61.3%)	43(10.5%)
I could afford to attend the health facility for treatment	178(43.5%)	120(29.3%)	76(18.5%)	35(8.5%)
I would advise my family to come to this facility for treatment if they had the same problem	21(5.1%)	45(11%)	124(30.3%)	219 (53.5%)

### Magnitude of patient satisfaction

The mean score of patient satisfaction among respondents who attend their treatment at Dilla university referral hospital, psychiatry clinic was 64 / 96, (95% CI, 61–67). By using the mean percentage transformation formula a continuous outcome variable which was the actual score of each respondent- minimum score of respondents (24)/maximum score of the respondents(96) -minimum score of the respondents(24)* 100. The final calculated result of all respondents was = 55.4% (95% CI (48.4%– 59.2%).

### Factors associated with patient satisfaction

During the multiple linear regression analysis at 95% CI and P value < 0.05, Having bipolar disorder diagnosis [β = -2.93, 95% CI (-4.33, -1.96), P = .000], distance from the hospital [β = -2.34), 95% CI (-3.765, -1.735), P = .001], waiting time [β = -2.19, 95% CI (- 3.49, -1.10) p = .000], monthly income (2.95, 95% CI (1.65, 5.23) and Urban residence (β = 1.43,95% CI (1.03–3.43), p = 0.01) were independent variables associated with patient satisfaction. The model fitness of the analysis (Adjusted square, R^2^) was = 0.75%) (Table 4).

**Table 4:** Linear regression analysis of respondents who attend their treatment at Dilla university referral hospital, Dilla, Southern Ethiopia, 2020, (n = 409).

		Simple linear regression		Multiple linear regression	
Variables	Category	Un-standardized Coefficients		Un-standardized Coefficients 95% CI	
		B	Sig	B	Sig
**Sex**	Male	-1.751 (-2.45–1.03)	0.341		
	Female	1			
**Age**	Age	0.123(-1.38–2.34)	0.459		
**Religion**	Orthodox	-0.231(-0.98–1.45)	0.562		
	Muslim	-0.193(0.74–1.18)	0.923		
	Protestant	1			
**Marital status**	Married	-3.12(-5.90–0.93)	0.471		
	Divorced	-0.827(-0.42–1.23)	0.273		
	Single	1			
**Educational status**	No-formal education	1			
	Primary	0.39(0.19–0.78)	0.123		
	Secondary	-2.13(-3.05–0.32)	0.343		
	> secondary	-3.34(-4.94–0.22)	0.453		
**Monthly income**	Income	3.14(1.56–4.89)	.003	2.95 (1.65–5.23)	0.02*
**Residence**	Urban	2.12(1.78–3.98)	0.001*	2.89 (1.23–6.34)	0.00**
	Rural			1	
**Age of onset illness**	Age at first onset of illness	-0.014(-149-0.99)	0.170*	-0.009(-0.025–0.007)	0.259
**Distance(km)**		-2.89(-3.93–0.21)	0.000*	-2.34(-3.76, -1.73)	0.00*
**Duration illness**	Total year	-.584 (-6.99–0.28)	0.430		
**History of Admission**	Yes	0.333(0.12–2.78)	0.323		
	No	1			
**Current Substance use**	Yes	-1.44 (-2.30–0.23)	0.321	-1.719	
	No			1	
**Waiting time (m)**	Waiting time	-3.34(-4.57–0.11)	0.000*	-2.19 (-5.23, -1.32)	0.001*
**Current psychiatry diagnosis**	BPD	-4.32(-5.78–0.13)	0.001	-2.93(-4.89, -2.43)	0.003*
	SCH	-2.12(-3.22–0.25)	0.09	-1.94(-2.13–0.22)	0.04
	MDD	1		1	
**Severity of illness**	Borderline mentally ill	0.899(0.29–2.88)	0.342		
	Mildly ill	-0.634(-0.89–1.22)	0.264		
	Normally ill	1			

(BPD, Bipolar disorder, MDD, Major depressive disorder, SCH, Schizophrenia)

* *Significant at p value <0*.*25*, during simple linear regression were selected for multi linear regression (1 = reference ***P< 0.001, ** P<0.01, * p<0.05, step wise analysis). Adjusted R2 = 0.75%.

## Discussion

The mean score of patient satisfaction in this study was 64/92, and the overall percentage score of patient satisfaction was 55.4% [95% CI, (46.3%– 58.4%)]. This study found a 55.4% overall percentage score of patient satisfaction which was lower than the study of Pakistan (92.7%) 95% CI (0.86–0.98) [15] and South Africa (72.9%) 95% CI (0.60–0.88) [14]. This discrepancy might be due to the difference in mental health literacy of respondents, setting of the mental health service, and availability of alternative mental health services. Also, this study finding was lower than that of the studies done in Mekelle (72%) [26], and Dessie (61.2%) [22]. This difference might be due to the difference in the assessment tool in which they used the Client satisfaction questionnaire (CSQ) and Clinical psychiatry outpatient satisfaction scale (CPOSS), respectively. It was also lower than the study done in Gondar (77%) 95% CI (0.70–0.84) [10] which might be explained by the difference in the setting of the service such as expert professionals, medication availability, Physical setup, and patient flow. This study found that 307 (75%) of respondents responded disagree, and strongly disagree with the item of referral to a specialist is possible, and this finding was contrary to the studies done in Jimma [9], 89% of them said agree and strongly agree to the item, and it might be due to lack of specialist and organized service on or nearby this study area.

This study found that more than two-thirds of the respondents reported dis-agree and strongly disagreeing with the items of the opportunity to have followed up with the same health worker and this study finding is supported by the studies done in Jimma [9] and Gondar [10]. It might be due to the random allocation of health care providers at outpatient and inpatient mental health services in most of the country.

This study found 294 (72%) of the respondents reported disagreeing and strongly disagree with the items of affordability for treatment, and supported by the studies done in Gondar [10] and Jimma [9], and This similarity could be owing to the high expense of medication, lack of accessibility at government health facilities, and a lack of funding from volunteer non-governmental organizations for long-term access to psychotropic medications in most parts of the country.

This study found that monthly income was positively associated with patient satisfaction (β = 2.95, 95% CI, (1.65, 5.23), P = 0.02), and this finding was supported by the study done in Addis Ababa [27]. The monthly income of respondents might have a direct association with the affordability of service, especially for transportation, routine baseline laboratory investigation, and buying medication.

This study found that living in urban areas was positively associated with patient satisfaction (β = 2.89, 95% CI, (1.23, 6.34), P = 0.00) and this study finding was contrary to the study done in Dessie [22], and Addis Ababa [27]. In urban areas, the vital things for getting the service such as transportation and medication are easily accessible.

This study found that distance was negatively associated with patient satisfaction (β = -2.34, 95% CI, (-3.76, -1.73), P = 0.00), and this study finding was supported by all the studies done in Addis Ababa [27], and Jimma [9]. Living a long distance from the hospital affects timely access to services, the ability to interact with physicians as needed, and respondents’ follow-up visits.

This study found that waiting time was negatively associated with patient satisfaction (β = -2.19, 95% CI, (-5.238,-1.321, P = 0.001), and this study finding has supported the studies done by Mekelle [26] and Jimma [9]. The time they spend in the hospital without receiving care has an impact on their ability to return home and work, as well as on their caregivers.

This study found that bipolar disorder was negatively associated with patient satisfaction (β = -2.93, 95% CI, (-4.890, -2.431), P = 0.003), and this study finding was the contrary to the studies done in Mekelle [26], Dessie [22]. It might be due to the high cost of mood stabilizer medications, especially Na-valproate, which is a highly costly, most effective, and preferred drug. Furthermore, the nature of the condition is episodic and relapsing, with three to four admissions each year. Moreover, patients require the safest and cleanest environment possible, which may be difficult in low-income countries.

## Conclusion

This study found that more than half of respondents were scored above the mean patient satisfaction score, and items that need modification were affordability, waiting time, referred by the specialist, and a chance to be followed by the same professional. Monthly income, living in an urban, distance from the hospital, diagnosis of bipolar disorder, and waiting time was negatively associated with patient satisfaction. Therefore working on the modifiable factors with responsible stakeholders is vital for enhancing patient satisfaction.

## Recommendation

There should be periodic evaluation and feedback about the level and predictors of patient satisfaction.

## Supporting information

S1 Data Minimal data set. (XLS)

## Abbreviations

CGIS: Clinical global impression scale
CI: Confidence interval
DURH: Dilla university referral hospital
ETB: Ethiopian birr
IRB: Institutional review board
MHSSS: Mental health service satisfaction scale
OPD: Out-patient Department
P-value: Probability value
SD: Standard deviation
SPSS: Statistical package for social science
